# 5-Chloro-1,3-dimethyl-1*H*-pyrazole-4-carbaldehyde

**DOI:** 10.1107/S1600536811041407

**Published:** 2011-10-12

**Authors:** Yong-Jun Shen, Mei Xu, Chong-Guang Fan

**Affiliations:** a College of Chemistry and Chemical Engineering, Nantong University, Nantong 226019, People’s Republic of China; b Department of Chemistry and Environmental Science, Cangzhou Normal University, Cangzhou 061001, People’s Republic of China

## Abstract

In the title compound, C_6_H_7_ClN_2_O, the mol­ecules are situated on mirror planes, so H atoms of two methyl groups were treated as rotationally disordered over two orientations each. The crystal packing exhibits weak inter­molecular C—H⋯O inter­actions and short Cl⋯N contacts of 3.046 (2) Å.

## Related literature

For the biological activity of pyrazole derivatives, see: Hamaguchi *et al.* (1995 ▶); Motoba *et al.* (1992 ▶). For a related structure, see: Yokoyama *et al.* (2004 ▶).
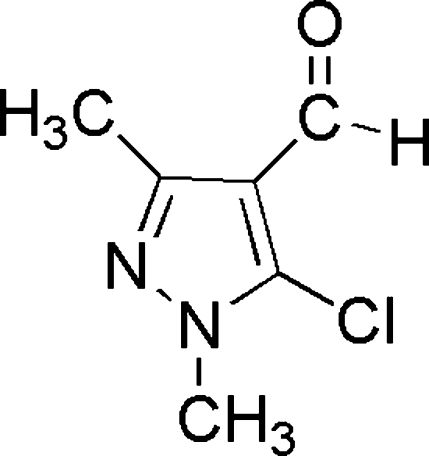


## Experimental

### 

#### Crystal data

C_6_H_7_ClN_2_O
*M* *_r_* = 158.59
Orthorhombic, 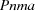

*a* = 13.167 (9) Å
*b* = 6.463 (5) Å
*c* = 8.190 (6) Å
*V* = 696.9 (8) Å^3^
*Z* = 4
Mo *K*α radiation
μ = 0.47 mm^−1^
*T* = 113 K
0.24 × 0.22 × 0.18 mm

#### Data collection

Rigaku Saturn724 CCD diffractometer
Absorption correction: multi-scan (*CrystalClear*; Rigaku, 2008 ▶) *T*_min_ = 0.895, *T*_max_ = 0.920
7166 measured reflections
897 independent reflections
726 reflections with *I* > 2σ(*I*)
*R*_int_ = 0.049

#### Refinement

*R*[*F* ^2^ > 2σ(*F* ^2^)] = 0.027
*wR*(*F* ^2^) = 0.081
*S* = 1.05
897 reflections
63 parameters
H-atom parameters constrained
Δρ_max_ = 0.36 e Å^−3^
Δρ_min_ = −0.25 e Å^−3^

### 

Data collection: *CrystalClear* (Rigaku, 2008 ▶); cell refinement: *CrystalClear*; data reduction: *CrystalClear*; program(s) used to solve structure: *SHELXS97* (Sheldrick, 2008 ▶); program(s) used to refine structure: *SHELXL97* (Sheldrick, 2008 ▶); molecular graphics: *SHELXTL* (Sheldrick, 2008 ▶); software used to prepare material for publication: *SHELXTL*.

## Supplementary Material

Crystal structure: contains datablock(s) global, I. DOI: 10.1107/S1600536811041407/cv5167sup1.cif

Structure factors: contains datablock(s) I. DOI: 10.1107/S1600536811041407/cv5167Isup2.hkl

Supplementary material file. DOI: 10.1107/S1600536811041407/cv5167Isup3.cml

Additional supplementary materials:  crystallographic information; 3D view; checkCIF report

## Figures and Tables

**Table 1:** Hydrogen-bond geometry (Å, °)

*D*—H⋯*A*	*D*—H	H⋯*A*	*D*⋯*A*	*D*—H⋯*A*
C5—H5*A*⋯O1^i^	0.98	2.58	3.220 (3)	123

Symmetry code: (i) 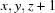.

**Fig. 1.**
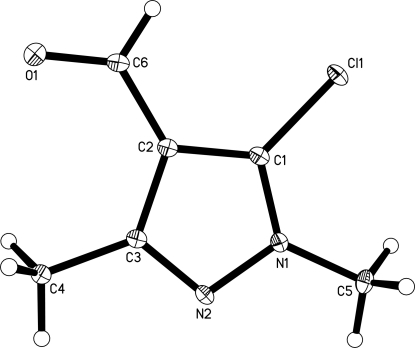
The molecular structure of (I) showing the atomic labels and 30% probability displacement ellipsoids.

## supplementary crystallographic information

### Comment

The pyrazole ring is a prominent heterocyclic scaffold in numerous bioactive molecules. Many pyrazole-based compounds are reported to possess diverse biological activities (Motoba *et al.*, 1992; Hamaguchi *et al.*, 1995). The title compound (I), is an important intermediate for the synthesis of agrochemicals and drugs. Details of its crystal structure may be helpful for the design of novel bioactive molecules.

In (I) (Fig. 1), all bond lengths and angles are normal and comparable with those observed in ethyl 4-formyl-1,3-dimethylpyrazole-5-carboxylate (Yokoyama *et al.*, 2004). All molecules in (I) are situated on mirror planes. The crystal packing exhibits weak intermolecular C—H···O interactions (Table 1) and short Cl···N contacts of 3.046 (2) Å.

### Experimental

To a well stirred cold solution of DMF(60 mmol) was added dropwise phosphoryl trichloride (90 mmol). The resulting mixture was stirred at 273 K for another 20 min. To the above solution was added 1,3-dimethyl- 1*H*-pyrazol-5(4*H*)-one (30 mmol), then it was heated to 363 k for 4 h. Completion of the reaction was checked by TLC, the reaction mixture was cooled and poured into cold water(100 ml). The pH of the mixture was adjusted to 7 by sodium hydroxide solution. The resulting solution was extracted with ethyl acetate (3 * 30 ml). The organic layer was dried over anhydrous sodium sulfate. The solvent was evaporated under reduced pressure, then the residue was recrystallized from ethyl acetate/petroleum ether to give a colourless crystal.

### Refinement

All H atoms were placed in calculated positions, with C–H = 0.95, and 0.98 ° A, and included in the final cycles of refinement using a riding model, with *U*_iso_(H) = 1.2*U*_eq_(C). H atoms of two methyl groups were treated as rotationally disordered over two orientations each with occupancies fixed to 0.5.

### Crystal data

**Table:**

C_6_H_7_ClN_2_O	*F*(000) = 328
*M**_r_* = 158.59	*D*_x_ = 1.511 Mg m^−^^3^
Orthorhombic, *P**n**m**a*	Mo *K*α radiation, λ = 0.71073 Å
Hall symbol: -P 2ac 2n	Cell parameters from 2460 reflections
*a* = 13.167 (9) Å	θ = 2.5–27.8°
*b* = 6.463 (5) Å	µ = 0.47 mm^−^^1^
*c* = 8.190 (6) Å	*T* = 113 K
*V* = 696.9 (8) Å^3^	Prism, colourless
*Z* = 4	0.24 × 0.22 × 0.18 mm

### Data collection

**Table:**

Rigaku Saturn724 CCD diffractometer	897 independent reflections
Radiation source: rotating anode	726 reflections with *I* > 2σ(*I*)
multilayer	*R*_int_ = 0.049
Detector resolution: 14.22 pixels mm^-1^	θ_max_ = 27.8°, θ_min_ = 2.9°
ω and φ scans	*h* = −16→17
Absorption correction: multi-scan (*CrystalClear*; Rigaku, 2008)	*k* = −8→8
*T*_min_ = 0.895, *T*_max_ = 0.920	*l* = −10→10
7166 measured reflections	

### Refinement

**Table:**

Refinement on *F*^2^	Primary atom site location: structure-invariant direct methods
Least-squares matrix: full	Secondary atom site location: difference Fourier map
*R*[*F*^2^ > 2σ(*F*^2^)] = 0.027	Hydrogen site location: inferred from neighbouring sites
*wR*(*F*^2^) = 0.081	H-atom parameters constrained
*S* = 1.05	*w* = 1/[σ^2^(*F*_o_^2^) + (0.0516*P*)^2^] where *P* = (*F*_o_^2^ + 2*F*_c_^2^)/3
897 reflections	(Δ/σ)_max_ = 0.002
63 parameters	Δρ_max_ = 0.36 e Å^−^^3^
0 restraints	Δρ_min_ = −0.25 e Å^−^^3^

### Special details

**Table:**

Geometry. All e.s.d.'s (except the e.s.d. in the dihedral angle between two l.s. planes) are estimated using the full covariance matrix. The cell e.s.d.'s are taken into account individually in the estimation of e.s.d.'s in distances, angles and torsion angles; correlations between e.s.d.'s in cell parameters are only used when they are defined by crystal symmetry. An approximate (isotropic) treatment of cell e.s.d.'s is used for estimating e.s.d.'s involving l.s. planes.
Refinement. Refinement of *F*^2^ against ALL reflections. The weighted *R*-factor *wR* and goodness of fit *S* are based on *F*^2^, conventional *R*-factors *R* are based on *F*, with *F* set to zero for negative *F*^2^. The threshold expression of *F*^2^ > σ(*F*^2^) is used only for calculating *R*-factors(gt) *etc*. and is not relevant to the choice of reflections for refinement. *R*-factors based on *F*^2^ are statistically about twice as large as those based on *F*, and *R*- factors based on ALL data will be even larger.

### Fractional atomic coordinates and isotropic or equivalent isotropic displacement parameters (Å^2^)

**Table:**

	*x*	*y*	*z*	*U*_iso_*/*U*_eq_	Occ. (<1)
Cl1	0.41884 (3)	0.2500	0.25597 (5)	0.01825 (17)	
O1	0.48840 (10)	0.2500	−0.28470 (15)	0.0222 (3)	
N1	0.62098 (12)	0.2500	0.23171 (16)	0.0159 (4)	
N2	0.70015 (10)	0.2500	0.12266 (17)	0.0166 (3)	
C1	0.53190 (11)	0.2500	0.1537 (2)	0.0146 (4)	
C2	0.54995 (11)	0.2500	−0.0124 (2)	0.0141 (4)	
C3	0.65799 (11)	0.2500	−0.0242 (2)	0.0142 (4)	
C4	0.72306 (11)	0.2500	−0.1739 (2)	0.0178 (4)	
H4A	0.7015	0.3620	−0.2468	0.027*	0.50
H4B	0.7161	0.1170	−0.2303	0.027*	0.50
H4C	0.7942	0.2710	−0.1426	0.027*	0.50
C5	0.63988 (14)	0.2500	0.4067 (2)	0.0234 (4)	
H5A	0.5928	0.3457	0.4603	0.035*	0.50
H5B	0.7099	0.2942	0.4276	0.035*	0.50
H5C	0.6297	0.1102	0.4500	0.035*	0.50
C6	0.47278 (12)	0.2500	−0.1387 (2)	0.0167 (4)	
H6	0.4039	0.2500	−0.1041	0.020*	

### Atomic displacement parameters (Å^2^)

**Table:**

	*U*^11^	*U*^22^	*U*^33^	*U*^12^	*U*^13^	*U*^23^
Cl1	0.0131 (3)	0.0215 (3)	0.0202 (3)	0.000	0.00588 (14)	0.000
O1	0.0198 (7)	0.0291 (8)	0.0177 (6)	0.000	−0.0013 (5)	0.000
N1	0.0138 (8)	0.0210 (8)	0.0130 (7)	0.000	0.0020 (5)	0.000
N2	0.0119 (7)	0.0226 (8)	0.0153 (7)	0.000	0.0035 (6)	0.000
C1	0.0119 (8)	0.0145 (9)	0.0175 (8)	0.000	0.0017 (6)	0.000
C2	0.0124 (8)	0.0137 (8)	0.0163 (8)	0.000	0.0001 (6)	0.000
C3	0.0125 (8)	0.0137 (9)	0.0163 (8)	0.000	0.0000 (6)	0.000
C4	0.0135 (8)	0.0239 (10)	0.0159 (8)	0.000	0.0008 (6)	0.000
C5	0.0237 (9)	0.0354 (12)	0.0111 (9)	0.000	0.0000 (7)	0.000
C6	0.0119 (8)	0.0182 (9)	0.0199 (8)	0.000	−0.0006 (7)	0.000

### Geometric parameters (Å, °)

**Table:**

Cl1—C1	1.7081 (18)	C3—C4	1.495 (2)
O1—C6	1.213 (2)	C4—H4A	0.9800
N1—C1	1.336 (2)	C4—H4B	0.9800
N1—N2	1.373 (2)	C4—H4C	0.9800
N1—C5	1.455 (2)	C5—H5A	0.9800
N2—C3	1.325 (2)	C5—H5B	0.9800
C1—C2	1.381 (2)	C5—H5C	0.9800
C2—C3	1.426 (2)	C6—H6	0.9500
C2—C6	1.450 (2)		
			
C1—N1—N2	110.83 (14)	C3—C4—H4B	109.5
C1—N1—C5	128.43 (15)	H4A—C4—H4B	109.5
N2—N1—C5	120.74 (15)	C3—C4—H4C	109.5
C3—N2—N1	105.82 (13)	H4A—C4—H4C	109.5
N1—C1—C2	108.67 (14)	H4B—C4—H4C	109.5
N1—C1—Cl1	122.06 (14)	N1—C5—H5A	109.5
C2—C1—Cl1	129.27 (13)	N1—C5—H5B	109.5
C1—C2—C3	103.80 (14)	H5A—C5—H5B	109.5
C1—C2—C6	125.60 (15)	N1—C5—H5C	109.5
C3—C2—C6	130.61 (15)	H5A—C5—H5C	109.5
N2—C3—C2	110.88 (14)	H5B—C5—H5C	109.5
N2—C3—C4	120.27 (14)	O1—C6—C2	125.74 (15)
C2—C3—C4	128.84 (15)	O1—C6—H6	117.1
C3—C4—H4A	109.5	C2—C6—H6	117.1
			
C1—N1—N2—C3	0.0	Cl1—C1—C2—C6	0.0
C5—N1—N2—C3	180.0	N1—N2—C3—C2	0.0
N2—N1—C1—C2	0.0	N1—N2—C3—C4	180.0
C5—N1—C1—C2	180.0	C1—C2—C3—N2	0.0
N2—N1—C1—Cl1	180.0	C6—C2—C3—N2	180.0
C5—N1—C1—Cl1	0.0	C1—C2—C3—C4	180.0
N1—C1—C2—C3	0.0	C6—C2—C3—C4	0.0
Cl1—C1—C2—C3	180.0	C1—C2—C6—O1	180.0
N1—C1—C2—C6	180.0	C3—C2—C6—O1	0.0

### Hydrogen-bond geometry (Å, °)

**Table:**

*D*—H···*A*	*D*—H	H···*A*	*D*···*A*	*D*—H···*A*
C5—H5A···O1^i^	0.98	2.58	3.220 (3)	123

Symmetry codes: (i) *x*, *y*, *z*+1.
